# APE1/Ref-1 as a Novel Target for Retinal Diseases

**DOI:** 10.33696/Signaling.2.044

**Published:** 2021

**Authors:** Curtis Heisel, Jonah Yousif, Mahmut Mijiti, Kostas Charizanis, Mitchel Brigell, Timothy W. Corson, Mark R. Kelley

**Affiliations:** 1 Herman B Wells Center for Pediatric Research, Department of Pediatrics, Indiana University School of Medicine, 1044 W. Walnut St., Indianapolis, IN 46202, USA; 2 University of Michigan Medical School, 1301 Catherine St, Ann Arbor, MI 48105, USA; 3 Kostas Charizanis, Independent Consultant, Livonia, MI 48150, USA; 4 Ocuphire Pharma Inc., Farmington Hills, MI, USA; 5 Department of Pharmacology and Toxicology, Indiana University School of Medicine, 1044 W. Walnut St., Indianapolis, IN 46202, USA; 6 Department of Biochemistry and Molecular Biology, Indiana University School of Medicine, 1044 W. Walnut St., Indianapolis, IN 46202, USA; 7 Indiana University Simon Comprehensive Cancer Center, Indiana University School of Medicine, 1044 W. Walnut St., Indianapolis, IN 46202, USA; 8 Eugene and Marilyn Glick Eye Institute, Department of Ophthalmology, Indiana University School of Medicine, 1044 W. Walnut St., Indianapolis, IN 46202, USA

**Keywords:** Redox effector factor 1, Apurinic/apyrimidinic endonuclease, Redox signaling, APE1/Ref-1, Angiogenesis, Inflammation, Transcription factors, Ocular clinical trial, Retina, Choroid, Neovascularization

## Abstract

APE1/Ref-1 (also called Ref-1) has been extensively studied for its role in DNA repair and reduction-oxidation (redox) signaling. The review titled: “*The multifunctional APE1 DNA repair-redox signaling protein as a drug target in human disease”* by Caston et. al. summarizes the molecular functions of Ref-1 and the role it plays in a number of diseases, with a specific focus on various types of cancer [1]. Previous studies have demonstrated that Ref-1 plays a critical role in regulating specific transcription factors (TFs) involved in a number of pathways, not only in cancer, but other disease indications as well. Disease indications of particular therapeutic interest include retinal vascular diseases such as diabetic retinopathy (DR), diabetic macular edema (DME), and neovascular age-related macular degeneration (nvAMD). While Ref-1 controls a number of TFs that are under redox regulation, three have been found to directly link cancer studies to retinal diseases; HIF-1α, NF-κB and STAT3. HIF-1α controls the expression of VEGF for angiogenesis while NF-κB and STAT3 regulate a number of known cytokines and factors involved in inflammation. These pathways are highly implicated and validated as major players in DR, DME and AMD. Therefore, findings in cancer studies for Ref-1 and its inhibition may be translated to these ocular diseases. This report discusses the path from cancer to the potential treatment of retinal disease, the Ref-1 redox signaling function as a possible target, and the current small molecules which have been identified to block this activity. One molecule, APX3330, is in clinical trials, while the others are in preclinical development. Inhibition of Ref-1 and its effects on inflammation and angiogenesis makes it a potential new therapeutic target for the treatment of retinal vascular diseases. This commentary summarizes the retinal-relevant research that built on the results summarized in the review by Caston et. al. [1].

## Introduction

### Ref-1 role in transcription factor (TF) regulation

**Figure 1:**
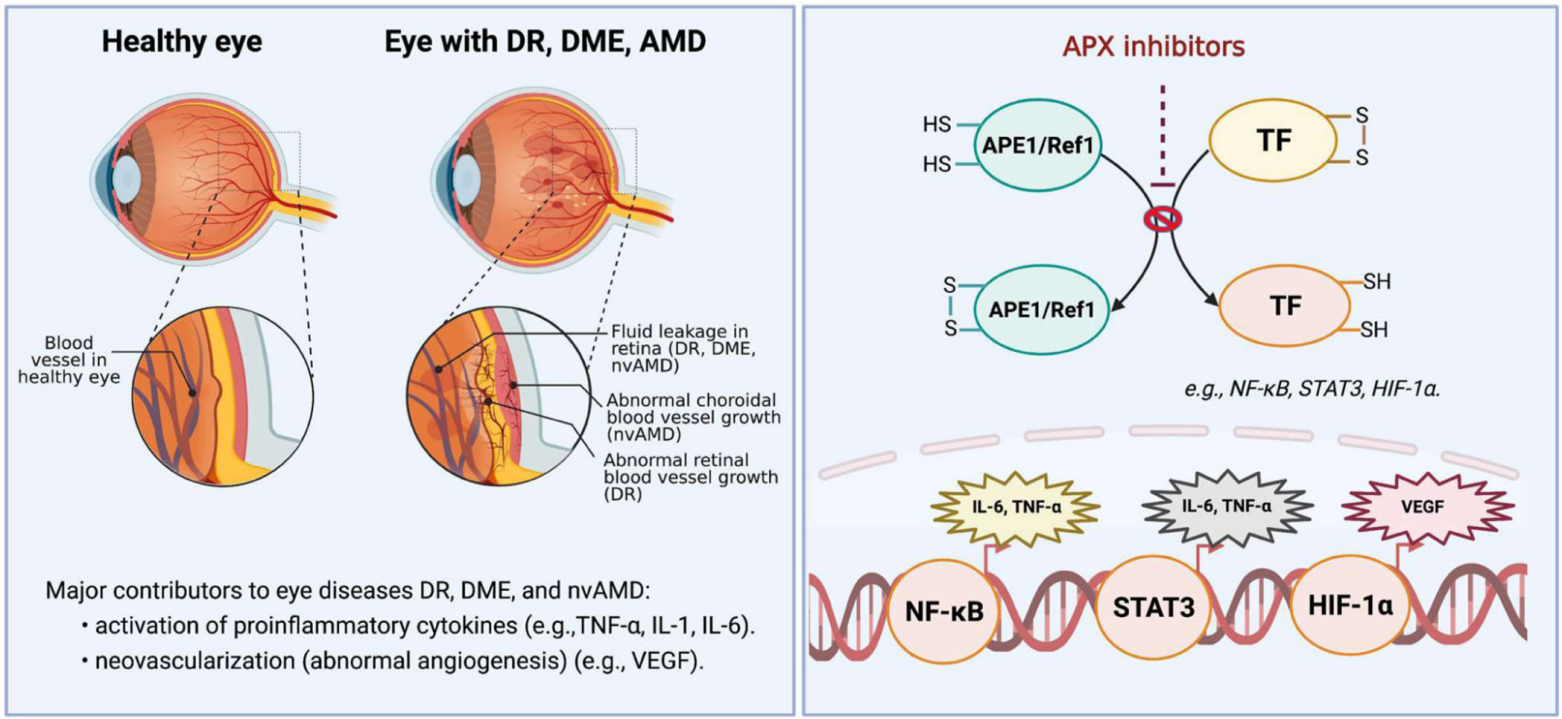
Role of Ref-1 in ocular diseases DR, DME, and nvAMD. Ref-1 plays a critical role in regulating specific transcription factors (TFs) involved in a number of pathways linked to retinal vascular disease such as HIF-1α, NF-κB and STAT3. HIF-1α controls the expression of VEGF that stimulates angiogenesis while NF-κB and STAT3 regulate a number of known cytokines and factors involved in inflammation. Both of these pathways are highly implicated and validated as major players in DR, DME, and nvAMD. APX inhibitors such as APX3330, currently in phase II trials for DR/DME and APX2009/APX2014 specifically block the ability of Ref-1 to convert inactive, oxidized TFs to active, reduced TFs. **Abbreviations**: TF(s): Transcription Factors; STAT3: Signal Transducer and Activator of Transcription 3; HIF-1α: subunit of Hypoxia-inducible factor 1; NF-κB: Nuclear Factor-κB; TNF-α: Tumor Necrosis Factor Alpha; IL-6: Interleukin 6; VEGF: Vascular Endothelial Growth Factor; DR: Diabetic Retinopathy; DME: Diabetic Macular Edema; nvAMD: Neovascular Age-related Macular Degeneration.

Extensive research has established Ref-1’s role in DNA repair and redox signaling [2–4]. As previously described, Ref-1 redox signaling is a highly regulated process that reduces oxidized cysteine residues in specific TFs as part of their activation [5–8]. The conversion of inactive, oxidized TFs such as HIF-1α, NF-κB, and STAT3 to active TFs through Ref-1 protein-protein interaction influences pathways involved with inflammation, cell growth, apoptosis, neuronal plasticity, and angiogenesis [5,6]. While these pathways have critical roles in the development of cancer, the angiogenesis and inflammation pathways are particularly relevant to retinal diseases such as DR, DME and nvAMD (Figure 1) [9, 10].

### Inhibiting Ref-1 redox signaling activity

Given the regulatory role of Ref-1 and its downstream pathways, research was initiated to determine whether the inhibition of Ref-1 might reduce processes like angiogenesis and inflammation. The compound APX3330 was originally developed by Eisai Co., Ltd. and Apexian Pharmaceuticals, Inc., as a selective Ref-1 inhibitor designed to treat chronic hepatitis C and B and cancer, as well as other disease indications [8]. APX3330 has been extensively characterized as a direct, highly selective inhibitor of Ref-1 redox activity that does not affect the protein’s other DNA repair endonuclease activity, not discussed here [1,8,11–15]. APX3330 is able to block Ref-1’s ability to convert various TFs from their oxidized, inactive state to an active, reduced state [16]. Furthermore, it selectively binds to both recombinant Ref-1 and Ref-1 from cell extracts, and displays additional specific, on-target properties [8,13,15,17,18]. Therefore, APX3330 is a specific inhibitor of Ref-1’s redox function [1,13].

Inhibiting Ref-1 activity with APX3330 in preclinical studies reduced angiogenesis and inflammation by decreasing transcription activation activity of NF-κB, HIF-1α, and increasing some downstream molecules they regulate, such as VEGF and inflammatory cytokines (e.g., interleukin-6 [IL-6], tumor necrosis factor alpha [TNF-α]) [6,11,19,20]. The regulation of NF-κB and HIF-1α are particularly relevant to angiogenesis and eye diseases such as DR, DME and nvAMD, as inflammation induced via NF-κB and HIF-1α driven VEGF expression are key mediators responsible for retinal and choroidal neovascularization (CNV) [10,21,22], the abnormal blood vessel growth patterns that are characteristic of proliferative DR and nvAMD, respectively. Moreover, VEGF is a major driver of the vascular leakage seen in DME [23].

## Ref-1 Inhibition: Expansion Beyond Cancer

As it became clearer that Ref-1 influences critical pathways beyond cancer, including those involved in retinal disease pathogenesis, research was initiated to characterize whether APX3330 might have a role in treatment of retinal vascular disease. A similar transition has been completed previously. Bevacizumab, also known as Avastin, was originally approved by the FDA to treat certain types of cancer [24]. As knowledge grew about its efficacy in treating retinal diseases, off-label use for these indications increased. APX3330 is similarly being studied for alternate indications but will eventually move towards its own FDA submission.

### Systemic Ref-1 inhibition with APX3330 can prevent laser-induced choroidal neovascularization

Research on the *in vivo* administration of APX3330 for the treatment of ocular neovascularization began with intravitreal delivery of the compound [10,21,25]. While this is the delivery route of the standard-of-care anti-VEGF biologics and ensures that the drug gets to the right place, in humans it is labor intensive, causes patient discomfort, and incurs a risk of potentially vision-threatening endophthalmitis [26]. As such, the systemic (intraperitoneal [i.p.]) administration of Ref-1 inhibitors was explored to evaluate if this could offer an alternative route of therapy for CNV [22]. Animals treated with APX3330 displayed significantly reduced CNV volume in a laser-induced mouse model as measured by fluorescent staining [22]. APX3330 was also tested in the laser-induced CNV model when administered by oral gavage, and was shown to effectively reduce L-CNV, as assessed *in vivo* by optical coherence tomography, and *ex vivo* by fluorescent staining of CNV. The decrease in lesion size demonstrated in this experiment was greater than 50%, which represented an increased reduction in lesion size from previous experiments in which APX3330 was administered by i.p. injection. This suggested that administering APX3330 through an oral route may be superior to i.p. injections for reducing CNV lesion size [27].

### Anti-inflammatory effects of Ref-1 inhibition with APX3330

The T1DM stroke model was also used to show the benefits of APX3330 in promoting healthy arterial density and improving function of macrophages. Influencing the behavior or presence of macrophages may change the local inflammatory process and vessel growth. Hypoxia-activated macrophages and microglia release TNF-α, which stimulates the release of IL-8, MCP-1, and VEGF in vascular cells or in retinal microglia cells. Such alterations in the endothelial cells of the retinal vessels lead to retinal ischemia, which stimulates the production of angiogenic factors such as VEGF and erythropoietin (EPO). APX3330 leads to reduction in expression levels of TNF-α, IL-6, and IL-12, partially explaining its anti-inflammatory effects [28]. Additionally, in adult human retinal pigment epithelium (RPE) cells, APX3330 reduced the transcriptional activity of NF-κB, a key factor associated with inflammation in angiogenesis. It also blocked activation of HIF-1α and reduced the expression of VEGF [21]. Additional studies have demonstrated that intrathecal injection of APX3330 decreased inflammation (*i.e.* reduced IL-6 expression) and alleviated pain, as assessed by measuring the paw withdrawal threshold with the von Frey test [29].

### APX3330 implications in neuronal protection

The mechanism of action of APX3330 and targeting Ref-1 also supports neuronal protection. This could offer an unexpected further benefit in DR and AMD if confirmed in retinal neurons, which are damaged in these diseases. Neuronal protection by APX3330 has been observed in dorsal root ganglion (DRG) and myenteric neurons [30–33]. While APX3330 is a targeted inhibitor of Ref-1’s redox function, additional data indicate that in the setting of neurons it can also enhance the DNA repair (Apurinic/Apyrimidinic [AP] endonuclease) activity of Ref-1, a major step in DNA base excision repair (BER). When isolated sensory neurons are exposed to APX3330, there is a concentration-dependent increase in Ref-1 endonuclease activity which is not observed in tumor cells [13,14,17,31].

Functionally, APX3330 protected sensory neurons from reactive oxygen species (ROS) production only in cells that had Ref-1 repair activity and not in cells in which the wild type Ref-1 was depleted and replaced with a Ref-1 mutant having only redox activity [31,34]. This is anticipated to translate, *in vivo*, to a protective mechanism whereby APX3330 facilitates BER to repair oxidative DNA damage and protect neurons, as it appears it is the DNA repair and not the redox function of Ref-1 which is necessary for sensory neuronal survival/function. Inflammation has also been shown to induce oxidative DNA damage and addition of APX3330 protects from this inflammation stress [30].

## Clinical Data Supporting Retinal Indications for Ref-1 Inhibitors

### APX3330 clinical safety

APX3330 has consistently demonstrated a favorable safety profile and tolerability profile across 11 trials at doses of up 600 mg/day. In five Phase II trials involving over 300 patients, adverse events occurred in <5% of the patients and at a similar rate between placebo and APX3330. In a sixth Phase I trial, two patients receiving 720 mg/day exhibited a diffuse skin rash that quickly reversed on reduction to a 600 mg/day dose. Four patients took 600 mg/day for over six months and three longer than 300 days without issue [35].

### APX3330 human pharmacokinetics

Human pharmacokinetics of APX3330 demonstrated plasma levels much greater than those seen in animals. Pharmacological studies with APX3330 in preclinical models demonstrated that, at a dose of 25 mg/kg, (equivalent to a 120 mg daily dose in humans), APX3330 achieved a concentration of 0.15–2 μg/mL in plasma. This plasma concentration was adequate to reach detectable levels in the retina and provide efficacy in reducing CNV. In support of these findings, APX3330 was detected in the eyes of mice even using a lesser dose of 10 mg/kg [27].

To refine the pharmacokinetic data for APX3330 in preparation for an ocular clinical trial, a physiological-based pharmacokinetic (PBPK) model was performed which predicted levels significantly higher than observed and required for efficacy in preclinical studies. At a 300 mg BID (600 mg/day total) dosing strategy, APX3330 is predicted to reach retinal concentrations of 15.4 μg/mL, which is ~85-fold greater retinal exposure than mice who were given APX3330 25 mg/kg (actual conc. in retina 0.176 μg/mL) [27]. These findings confirm the 300 mg dose BID (total 600 mg/day) of APX3330 in the ZETA-1 Phase II trial sponsored by Ocuphire Pharma.

### Current clinical trial status of APX3330

Over a decade of research has characterized APX3330 as a potential therapeutic option for retinal and choroidal vascular disease. The ZETA-1 trial is a randomized, placebo-controlled, double-masked Phase 2 study designed to evaluate the efficacy of APX3330 to improve diabetic retinopathy over 24 weeks. The study will be conducted in up to 20 U.S. sites and is expected to enroll approximately 100 subjects with moderately-severe to severe non-proliferative diabetic retinopathy (NPDR) or mild proliferative diabetic retinopathy (PDR) in the study eye. If patients who are enrolled also have DME in their non-study eye, this eye will also be followed during the trial for potential improvement. The primary endpoint of the study will evaluate the percentage of subjects with a ≥ 2 step improvement on the Diabetic Retinopathy Severity Scale (DRSS) score. Secondary endpoints include evaluation of central subfield thickness to assess effects on diabetic macular edema, BCVA, safety and tolerability. If successful, the ZETA-1 trial will further advance the research done on Ref-1 and APX3330, and advance the drug toward providing the first oral option for DR as well as an adjunct therapy that may improve dosing convenience and compliance by alleviating some of the burden of chronic anti-VEGF injection treatments for DME and other retinal diseases such as nvAMD.

## Future Directions

In addition to DR, APX3330 is being explored for treatment of DME and nvAMD. Furthermore, there is a robust pipeline of APX3330 analogues such as APX2009 and APX2014 [1,22] that can also be explored for multiple delivery routes for multiple ocular indications, and potentially used in combination with currently approved drugs, such as the anti-VEGF agents aflibercept or bevacizumab [22]. Given the important role of Ref-1 in pathways involved in retinal disease, there is great potential in targeting it as a novel therapeutic option.

## Abbreviations:

TF: Transcription Factor
DR: Diabetic Retinopathy
DME: Diabetic Macular Edema
nvAMD: Neovascular Age-related Macular Degeneration
VEGF: Vascular Endothelial Growth Factor
CNV: Choroidal Neovascularization
i.p.: intraperitoneal
TNF-α: Tumor Necrosis Factor Alpha
EPO: Erythropoietin
RPE: Retinal Pigment Epithelium
DRG: Dorsal Root Ganglion
BER: Base Excision Repair
ROS: Reactive Oxygen Species
PBPK: Physiological-Based Pharmacokinetic
NPDR: Non-Proliferative Diabetic Retinopathy
PDR: Proliferative Diabetic Retinopathy
DRSS: Diabetic Retinopathy Severity Scale
